# Designing citizen science tools for learning: lessons learnt from the iterative development of nQuire

**DOI:** 10.1186/s41039-018-0072-1

**Published:** 2018-05-11

**Authors:** Christothea Herodotou, Maria Aristeidou, Mike Sharples, Eileen Scanlon

**Affiliations:** The Open University, Walton Hall, Milton Keynes, Buckinghamshire MK7 6AA UK

## Abstract

This paper reports on a 4-year research and development case study about the design of citizen science tools for inquiry learning. It details the process of iterative pedagogy-led design and evaluation of the nQuire toolkit, a set of web-based and mobile tools scaffolding the creation of online citizen science investigations. The design involved an expert review of inquiry learning and citizen science, combined with user experience studies involving more than 200 users. These have informed a concept that we have termed ‘citizen inquiry’, which engages members of the public alongside scientists in setting up, running, managing or contributing to citizen science projects with a main aim of learning about the scientific method through doing science by interaction with others. A design-based research (DBR) methodology was adopted for the iterative design and evaluation of citizen science tools. DBR was focused on the refinement of a central concept, ‘citizen inquiry’, by exploring how it can be instantiated in educational technologies and interventions. The empirical evaluation and iteration of technologies involved three design experiments with end users, user interviews, and insights from pedagogy and user experience experts. Evidence from the iterative development of nQuire led to the production of a set of interaction design principles that aim to guide the development of online, learning-centred, citizen science projects. Eight design guidelines are proposed: users as producers of knowledge, topics before tools, mobile affordances, scaffolds to the process of scientific inquiry, learning by doing as key message, being part of a community as key message, every visit brings a reward, and value users and their time.

## Introduction

This paper presents a set of interaction design principles to guide the development of citizen science tools that have an explicit focus on citizens’ or volunteers’ learning. The principles were produced through a 4-year iterative process of design, development, and evaluation with 240 end users, a group of eight experts in technology-enhanced pedagogy and user experience, and seven structured interviews with citizen science volunteers. A design-based research (DBR) methodology (Edelson 2002) guided the design process, yet with a focus on the refinement of a central, theoretical concept, that of ‘citizen inquiry’, and its instantiation in educational technologies, rather than the production of domain theories, design frameworks, or design methodologies. Citizen inquiry (Herodotou, Sharples, Scanlon, 2018) is the concept behind the development of the proposed citizen science tools. This concept was generated from earlier empirical work in the fields of citizen science and inquiry learning, in particular work on technology-enabled inquiry learning as summarised in the edited book Orchestrating Inquiry Learning (Littleton et al. 2012) and involvement in designing citizen science platforms such as iSpot (Silvertown et al. 2015). Specifically, research was previously carried out in ‘personally meaningful science investigations’ (through the Personal Inquiry project). The focus of that work was on science investigations of relevance to young people, guided by a teacher, and starting in formal education settings, that of the classroom. This line of research was further developed to understand how such guided discovery approach could be extended to the wider public, by exploring how citizen science might be a basis for personally meaningful inquiry learning.

The central idea of citizen inquiry is the engagement of the general public in proposing, designing, managing, analysing, and sharing scientific investigations. The underlying assumption, which is yet to be tested, is that engagement in all the stages of a scientific investigation will enable citizen science volunteers to gain insight into the practices and challenges of science investigation, while also contribute useful scientific knowledge in topics of personal interest (Herodotou, Sharples, Scanlon, 2018). Citizen inquiry has potential to alter the relationship that most people have with scientific research from consumption to one of active engagement (Sharples et al. 2013, p. 5). As detailed in the first edited collection (Herodotou, Sharples, Scanlon, 2018), citizen inquiry connects inquiry learning and citizen science, as an innovative inquiry learning approach to be used in teaching and learning, and one to attract interest and engage the general public in research-led endeavours. The role of scientists is to share their knowledge and skills with volunteers and support the implementation of citizen-led projects, thus reversing existing relationships in citizen science projects where the general public contributes to projects initiated and managed by professional scientists. Members of the public can gain direct experience of acting as scientists, and scientists may educate the wider public in practices of scientific inquiry. Thinking critically is an integral part of the scientific inquiry and of particular value in the digital era; the general public has access to diverse sources of information on the web that are not regulated or accurate. It becomes the users’ responsibility to judge the quality of information and decide on its validity, raising the need for learning approaches that can empower people to critically engage with online resources and conclude on their correctness. Towards this end, citizen inquiry tools can scaffold the development of critical thinking skills through engagement with the various stages of the scientific inquiry and communication with others, including experts and the broader community. In the next sections, we indicate how theory grounded in the concept of citizen inquiry, pedagogy-led expertise, and user experience studies informed each other and led to the design of a set of citizen science tools for supporting inquiry learning, in particular the nQuire-it platform and the Sense-it mobile application.

## A design-based research approach to citizen science and learning

### Citizen science

Citizen science refers to the participation of members of the public in research-led activities, such as species recognition (e.g. invasive species) or galaxy classification (Toerpe 2013). Inherent in the definition of citizen science is the notion of volunteering and dedication of the public to practices of science. Also called crowd-sourced science, citizen science is emerging as ‘the favoured twenty-first century model for conducting large-scale scientific research’ (Toerpe 2013). As Silvertown (2009) explains, the two main reasons driving the growth of citizen science are availability of technical tools for analysing the large volumes of data collected and realisation of the power behind this paradigm; the public comprises a free source of labour and skills that can overcome the financial and logistical constraints required for doing large-scale science (Catlin-Groves 2012). The benefits of citizen science endeavours can be both scientific and educational. Benefits to science include ‘large spatial scales, long time series, data from private land, and labour-intensive data that would otherwise be expensive to collect’ (Freitag and Pfeffer 2013, p. 1). The educational and social benefits include ‘educating the public in science and scientific thinking, inspiring appreciation of nature, and promoting support for conservation initiatives’ (Freitag and Pfeffer 2013, p.1). Evidence suggest enhanced learning outcomes over time, in terms of increasing accuracy and degree of self-correction of observations (Bonney et al. 2009). Participation in citizen science projects could contribute to the demand for proficiency in science, technology, and mathematics, by offering hands-on opportunities to amateurs and boosting their interest in these disciplines (Toerpe 2013).

Despite its scientific and educational merits, citizen science faces a number of challenges. First, most citizen science projects recruit people as data collectors or analysts rather than engaging them in all aspects of the scientific process. The frequently adopted model for doing citizen science is a ‘top-down’ one, with volunteers acting at a distance as data collectors. As Mueller et al. (Mueller et al. 2012, p. 3) describe it, ‘very seldom do citizens actually witness a scientist in action’. Some projects engage the public more closely with scientists, by generating a top-down program of investigation, then asking citizens to propose new questions or to challenge existing methods and approaches (Shirk et al. 2012). Having non-professionals devise their own scientific questions and activities is a demanding task, for proposed investigations should be personally relevant, accomplished using recognised methods of data collection and analysis, and be valid and ethical (Villasclaras-Fernandez et al. 2013). Another challenge citizen science faces is young people’s participation in citizen science activities. The preliminary demographic analysis makes reference to predominantly middle income and age citizens, with a great proportion of them being retirees (Toerpe 2013; Cornwell 2011). A middle-aged group of the public takes part in citizen science projects (Catlin-Groves 2012, p. 11), so there is a need for projects that will attract younger participants and diversify participation. Projects that involve personal technologies and social networks may offer a way to facilitate engagement and encourage the participation of younger people (Newman et al. 2012). With 81% of UK teens owning a smartphone (eMarketer 2014), identifying ways to merge mobile phone use and citizen science projects might effectively address this challenge and attract young people’s interest in citizen science.

### Inquiry learning

Inquiry-based learning involves learners in posing questions about the natural and material world, collecting and analysing data to identify responses to their queries, and making and testing hypotheses (de Jong 2006). It can be a powerful means for gaining knowledge about the natural and social world (de Jong 2006) and developing similar thinking competences to those of scientists (Edelson et al. 1999). A recent approach to inquiry learning emphasises the involvement of learners in devising personally meaningful scientific investigations by setting research questions that match their interests, defining and carrying out their own methodological design, collecting and analysing data, and sharing and reflecting on their research (Anastopoulou et al. 2012). Being a problem-based context featuring complex and difficult tasks, inquiry learning requires scaffolding to make tasks manageable, support learners’ understanding, and encourage self-expression and reflection (Quintana et al. 2004). This guidance can be distributed across teaching material including educational software, teachers and mentors, and learners themselves (Puntambekar and Kolodner 2005). Structured scaffolding, guidance, and coaching facilitate cognitive apprenticeship, enhancing learners’ problem-solving skills (Quintana et al. 2004), and decreasing cognitive load by drawing learners’ attention to aspects of the task that are relevant to learning goals (Hmelo-Silver 2006). The Personal Inquiry project—a collaborative research project conducted by The Open University and the University of Nottingham (2007–2010)—scaffolded personal inquiry through ‘scripts’ in the form of computer software that implements learning activities and lesson guides and engaged young people (aged 11–16) in carrying out scientific inquiries supported by teachers and resourced by a personal inquiry toolkit. Learners investigated personally meaningful issues related to their lives and interests, such as healthy eating, through a scientific process of inquiry. The design of the nQuire web-based platform enabled the implementation of inquiry-based learning activities distributed across formal and informal settings including the classroom, home, and discovery centres. The nQuire-it toolkit presented in this paper is a redevelopment of the original nQuire software, moving away from the school setting to scaffold citizen inquiry and untutored informal learning.

Other major projects that support inquiry learning are the Web-based Inquiry Science Environment (WISE) (see https://wise.berkeley.edu/) and the Global Online Science Labs for Inquiry Learning at School (Go-Lab) (www.go-lab-project.eu/). WISE is designed to support science teachers and K-12 students. It provides an online platform with features and tools that promote inquiry, such as reading and writing prompts, argument organisers and explanation generation tools, activity templates, rich media and interactive simulations, monitoring and engaging with students, customising curricula authoring features, and grading and feedback tools. The combination of the above in a virtual learning environment intends to enhance students’ experience of exploring new ideas and evidence while engaging in collaborative reflection supported by teacher’s feedback. Go-Lab aims to engage students, aged 10–18, in scientific inquiry and facilitate them in acquiring inquiry skills and experience in doing science through guided experimentation. Teachers create their own inquiry learning space and customise it according to their needs, by adding instructions, educational resources, and exercises. The learning space is supported by an inquiry learning cycle which guides the students through the phases of inquiry (formulating research questions, conducting experiments, drawing conclusions). Go-Lab also integrates a community and tutoring platform for collaboration and practice sharing with other teachers and experts. These projects are school science and curriculum oriented and do not engage people of all ages and abilities in initiating and conducting science investigations.

Citizen science projects such as Galaxy Zoo (www.galaxyzoo.org) may involve citizens in specific elements of the inquiry learning process such as observation and classification of objects. The research objectives and methods of organising the research endeavour are defined by scientists, with the risk of only engaging those members of the public whose interests align well with the project’s aims. By contrast, web-based project initiation platforms, such as Kickstarter (www.kickstarter.com), allow people to propose and organise personally meaningful projects for raising funds but do not provide a model of scientific inquiry.

### Citizen inquiry

Citizen inquiry combines the knowledge development of inquiry-based learning with the research paradigm of citizen science, to engage citizens with diverse interests and motives in all the stages of the scientific process of inquiry: conception of a project, definition of research objectives, selection of methods of data collection and analysis, and implementation of research. It is proposed as an innovative approach to inquiry learning that draws from the benefits of citizen science and mass participation to provide opportunities to the general public, and not only scientists, to experience science, act as scientists, and learn the scientific method through ongoing communication with others. Citizen inquiry is proposed as a new approach to teaching and learning that overcomes the boundaries of natural sciences, the roots of citizen science, and applies to diverse domains and disciplines such as social sciences, humanities, and psychology (Herodotou, Sharples, Scanlon, 2018). The conceptual understanding of citizen science as a new approach to learning and one that is applicable to the general public, and not only scientists, underpinned our design of citizen science tools for learning. Also, it defined the technological affordances of the tools by incorporating functionality to support project initiation by volunteers and mechanisms for setting up different types of projects (see the ‘Findings from the first design iteration’ section). As a result, we designed tools that could engage volunteers in creating their own personally meaningful investigations (Design principle 1). In addition, considering for the reported diversity of interests and motivations across volunteers in citizen science projects (e.g. Curtis 2015), we also designed tools that could support the creation of diverse (types and topics) investigations ensuring that volunteers with different orientations could join citizen inquiry activities (Design principle 2)

*Design principle 1 (theory): Engage volunteers as initiators of citizen science projects, to create personally relevant investigations.*

*Design principle 2 (theory): Vary the investigations (types and topics) to accommodate different interests and motivations of volunteers who participate in citizen science projects.*

## Methods

### Preliminary work

The generation of the concept of citizen inquiry has led us to explore the fields of citizen science and inquiry learning to identify existing design spaces that might accommodate and support the design of a citizen inquiry web-based platform. We reviewed the design of citizen science websites such as Zooniverse, Kickstarter, and project Noah (www.projectnoah.org) and gathered elements that could contribute to engaging the public with citizen inquiry. For example, the thematic organisation and presentation of projects in Zooniverse (www.zooniverse.org) and Kickstarter (www.kickstarter.com) offered insights (e.g. design ideas, scaffolding mechanisms) into how we could organise and present crowd-sourced investigations in a citizen inquiry website. The project Noah (www.projectnoah.org) showed how we could personalise the citizen inquiry experience through the creation of customised profile page showing users’ participation in different projects and presentation of personal information. Also, we reviewed previous work on inquiry learning, in particular the design of the web platform nQuire (Anastopoulou et al. 2012). The platform was specifically designed to scaffold students’ inquiry learning across formal and informal settings. Teachers and students could set up their own inquiry learning investigations. The platform guided students through all the stages of inquiry learning from setting their research objectives, devising a methodology, collecting and analysing data, and reaching a conclusion. The online implementation of the process of inquiry learning on the nQuire platform provided insights into the types of interaction with the platform and how inquiry learning could be successfully supported online. Also, it indicated limitations of nQuire as a platform for citizen inquiry, since its design was based on the assumption that a teacher with expertise in inquiry-led learning would guide the investigations. Each investigation on nQuire followed a visible structure that guided learners through the stages of scientific method. The platform is designed to support groups assigned by a teacher, rather than attract recruits through social media. These considerations led to the production of four different sets of mock-ups visualising the design and interactions of the citizen inquiry platform.

The four sets of mock-ups were presented and discussed in the academic working group responsible for the design of the citizen inquiry platform. This group consisted of a software developer, a research associate, and a member of the academic staff. The strengths and weaknesses of the four mock-ups were identified. The ultimate aim was to design a functional and user-friendly platform that would support social interaction and communication in order for young people to get attracted to it and start using it. Thus, the mock-ups focused on simplifying the visual presentation and creating the social mechanisms that were not provided in the previous nQuire platform. The designs of the mock-ups were closely related to the theoretical conceptualization of citizen inquiry. They allowed for the creation of, or participation in, different types of projects. They suggested a simple process of setting up a project and provided scaffolds as to how to initiate a new investigation, in order to engage the general public with less or no science knowledge. They featured connections to popular social media to share projects and identify others to take part in the proposed investigations. They provided example investigations to engage citizens with diverse interests and motives in the scientific process of inquiry. They also followed principles of effective online learning, including making the learning goals and processes visible (Hattie 2009) through labelling and titling of the investigations, and profiles of users. They enabled learning as a social and collaborative process by integrating with social network sites. They supported conversations for learning (Pask 1975; Laurillard 2002) by associating user comments and discussions with each element of the inquiry.

### Overview of design-based research

The nQuire-it toolkit is the concrete manifestation of the concept design which contributed to the theoretical development of the concept of citizen inquiry. It emerged from a combination of theory-informed design and the critique of the mock-ups. It consists of the nQuire-it Missions website (www.nquire-it.org.uk) and the Sense-it app for Android mobile devices (available in Google Play). nQuire-it is a web-based platform that hosts the development and management of citizen inquiry investigations. It is a test site to trial citizen inquiry methods and tools. Sense-it, a sensor-based mobile application, is connected to the platform to support data collection using mobile devices. The nQuire-it toolkit is the outcome of the nQuire: Young Citizen Inquiry project, a 1-year research and development project funded by the Nominet Trust and coordinated by The Open University (September 2013 to August 2014). The project aimed to design and develop a set of open-access tools for scientific investigation in collaboration with young people, to support young people aged 15–19 in engaging with the tools and investigations and to implement cycles of design, evaluation, and reporting, through a sequence of workshop activities with the Sheffield University Technical College (UTC) and the young user community online. Subsequently, in year 2 of the project, the scope of nQuire-it was extended to adult participants through a study with amateur meteorologists and weather watchers conducted by one of the authors (author 2) as part of her Ph.D. research (reference removed). In years 3 and 4 of the project, the research team partnered with the BBC Tomorrow’s World initiative (http://www.bbc.co.uk/tomorrowsworld) to extend the nQuire-it website and tools to support large-scale public experiments in psychology and social science. For example, members of the public will be able to create and run online studies to explore attitudes and personality. This will involve providing new ways to enter responses, secure handling of personal data, and ways for participants to see overview results. This work also involves re-implementing nQuire-it to run large studies linked to BBC TV or radio programmes.

## Results

### First design iteration with end users

Three design experiments were run to evaluate and improve the concept design. The first design experiment was run with students (*N* = 96, aged 16–18) and staff from Sheffield University Technical College (UTC), a school for students aged 14–19 that combines technical, academic, and practical learning. Aligning with the pragmatic character of design-based research (Wang and Hannafin 2005), we initiated the research by asking UTC staff to identify and suggest areas where the design of a citizen inquiry tool would complement their teaching and make a contribution to science learning and understanding, in particular students’ participation in inquiry learning. UTC staff proposed the design of a sensor-based mobile application as a suitable tool for running science inquiries and facilitating understanding of science topics such as physics and engineering. This proposal to harness the power of sensors on mobile devices challenged our initial assumptions of the concept design, as well as the actual design of artefacts, and emphasised how mobile devices may engage young people with citizen inquiry. This insight led to revisions of the concept and design, leading to the development of prototypes of the nQuire-it Missions website and Sense-it mobile app. These prototypes were evaluated by experts in human-computer interaction using the method of heuristic usability evaluation (Molich and Nielsen 1990).

The aims of the first design experiment were (a) to identify design aspects that require further improvement, (2) to identify citizen inquiries that could be hosted on the platform, (3) to identify potential issues that inhibit users from joining an inquiry proposed by other people, and (4) to investigate how to create a sustainable online community for citizen inquiry. It required students to evaluate the Sense-it prototype in order to improve its design and propose citizen inquiries using the tool. Students formed 14 groups. They were then allocated mobile devices with the Sense-it app and asked to test the app and complete a set of evaluation worksheets. Worksheet 1 asked students to propose two science investigations using the app by writing down a title, a specific question, and how to use the app to collect data. Worksheet 2 asked them to write down what they like the most about the app, what they like the least about it, and what they would like to change on it. Outcomes from this evaluation led to design improvements.

The second design experiment was also run with Sheffield students (*N* = 43, aged 16–18). A more improved version of the Sense-it app and nQuire-it platform was presented to the students. In the first activity, students in groups were asked to explore the nQuire-it platform and complete a worksheet about what they like/dislike about the tools and how they would improve their design. In the second activity, they were asked to create their own mission, make it public on the platform and complete a worksheet with the following questions: (1) Explain what your mission was about; (2) What difficulties/problems did you find when trying to create a mission? and (3) What would you like to change or add to the platform or app that would make a mission more useful or more fun? In the final activity, students were asked to take part and evaluate a mission proposed by another group of students and state their degree of agreement with a set of statements (5-point Likert scale) that examined their understanding and satisfaction with the mission they joined.

The third design experiment was run for 14 weeks with adult volunteers (*N* = 101). Participants were recruited for the ‘Weather-it’ project, an online inquiry investigation about weather. Participants varied in weather expertise (experts, novices) and were asked to create their own and/or join weather-related missions on the nQuire-it platform. Missions created referred to everyday life weather questions, weather phenomena of personal interest, and climate-related inquiries. Participants, in particular novices, received support by a moderator (author 2) when designing their own missions. Expert participants did not request any support, yet they were voluntarily contributing their expertise to other missions. The aim of the third experiment was to capture participants’ engagement with Weather-it investigations using Social Network Analysis (SNS) and identify how best to create and sustain a citizen inquiry community.

### Findings from the first design iteration

The outcomes from the three design experiments led to concept revisions, design of an improved user experience, and the generation of a set of design principles to guide the creation of citizen inquiry projects. Data from the first design experiment about the Sense-it app were analysed using thematic analysis. Student responses were clustered based on meaning and reduced into summary categories. The analysis revealed the following themes: (a) student satisfaction: students were found to be satisfied by the potential to customise sensors, the fact that the recording data were presented in two formats (numbers and graphs) and could be saved; (b) suggestions for improvements: accessibility issues including the complex information display on graphs and sensors, difficulty in navigation, the need for simplicity, and attractiveness in terms of colours, sensor icons design, and fonts; and (c) proposed science investigations: these were grouped into sound, light, acceleration, and temperature such as ‘What is the acceleration and top speed of the lifts in UK?’ (Herodotou, Villasclaras Fernandez, Sharples, 2014a). Participants’ active involvement in initiating science investigations was an opportunity for members of the public to define and pursue personally meaningful investigations rather than contribute only to data collection practices defined by scientists.

*Design principle 3 (user experience): Develop mobile applications to scaffold data collection citizen inquiry projects.*

In the second design experiment, students’ responses were thematically analysed in aspects of the tools students liked, aspects of the tools they did not like, and suggestions for improvements. Students were found to be (a) satisfied with the interface of the nQuire-it platform (colours, layout, logo, and pictures), navigational simplicity, and the information they derived from it; (b) less satisfied with unfinished aspects of the platform such as the logging in with social network accounts and difficulties in understanding graphs uploaded from the Sense-it app; (c) participation in missions: students faced difficulties related to technical issues, such as how to record data, and mission implementation, such as how to edit the mission; (d) suggestions for improvements related to the addition of more and varied missions, guidance and explanations on what they might do with it, and social aspects such as a rating system, updates from other members, and a chat room; and (e) examples of missions created are (1) how much do you move when you sleep? (2) computer loudness test: which computer is louder? and (3) find the noisiest UK ponds.

The missions created by students all had a title and a very brief description of what the mission was about. However, the descriptions were short, with no details on the rationale behind each mission. In terms of the process of data collection, none of the missions gave specific details as to how data should be collected. This might indicate that students lacked the understanding and skills needed to devise valid methods for data collection (e.g. what tools to use, how to use them). This assumption is further supported by the fact that some of the proposed missions were not feasible (e.g. ‘Where does a compass point in space?’).

The students found it easy to join a mission created by another person or group, yet hard to understand how to run it, as tasks were not clearly defined. Therefore, they proposed changes related to how the mission is explained and presented and how data are recorded. Students’ responses to a 5-point Likert scale indicated that the missions’ objectives were understandable (M = 4.14, SD = 1.02). However, the information about taking part in a mission (M = 3.7, SD = 1.11), the specificity of instructions (M = 3.4, SD = 1.5), and feasibility of the mission (M = 3.7, SD = 1.6) were less clear. Also, while it was not difficult to use the Sense-it app (M = 1.2, SD = .7), they faced problems in uploading (M = 2.7, SD = 2.1) and identifying their data on the platform (M = 3.0, SD = 1.7) and understanding its meaning (M = 2.6, SD = 1.9). In terms of the meaningfulness of the mission they took part in, their responses were moderate (M = 3.7, SD = 1.1). The second design experiment stressed the need for a simple interaction interface and appropriate scaffolds to support the process of data collection, spark ideas for science investigations that are feasible, and guide users through the process of participating or initiating a citizen inquiry.

*Design principle 4a (user experience): Scaffold the process of scientific inquiry from setting up a new investigation, to choosing tools for data collection or contributing to investigations set by others.*

In the third design experiment, social network analysis (SNS) was used to visualise the interactions amongst participants in weather investigations (*N* = 78). SNS demonstrated the structure of the interactions, the participation of members, the linking between missions (Aristeidou, M., Scanlon, E., Sharples, M., 2015a), and helped estimate how the community evolved over time and which reinforcement activities prompted that evolution (Aristeidou, M., Scanlon, E., Sharples, M., 2015b). The level of participation and contribution to missions varied due to participants’ diverse individual interests. Also, none of the proposed types of the mission was found to be dominant (see Fig. 4). The level of participation largely depended on the facilitation activities by the moderator. In particular, the establishment of weekly email updates and email notification system increased and sustained participants’ activity for the duration of the design experiment (14 weeks).

*Design principle 4b (user experience): Moderate interactions and facilitate participation through a set of mechanisms such as weekly email notifications.*

**Fig. 1:**
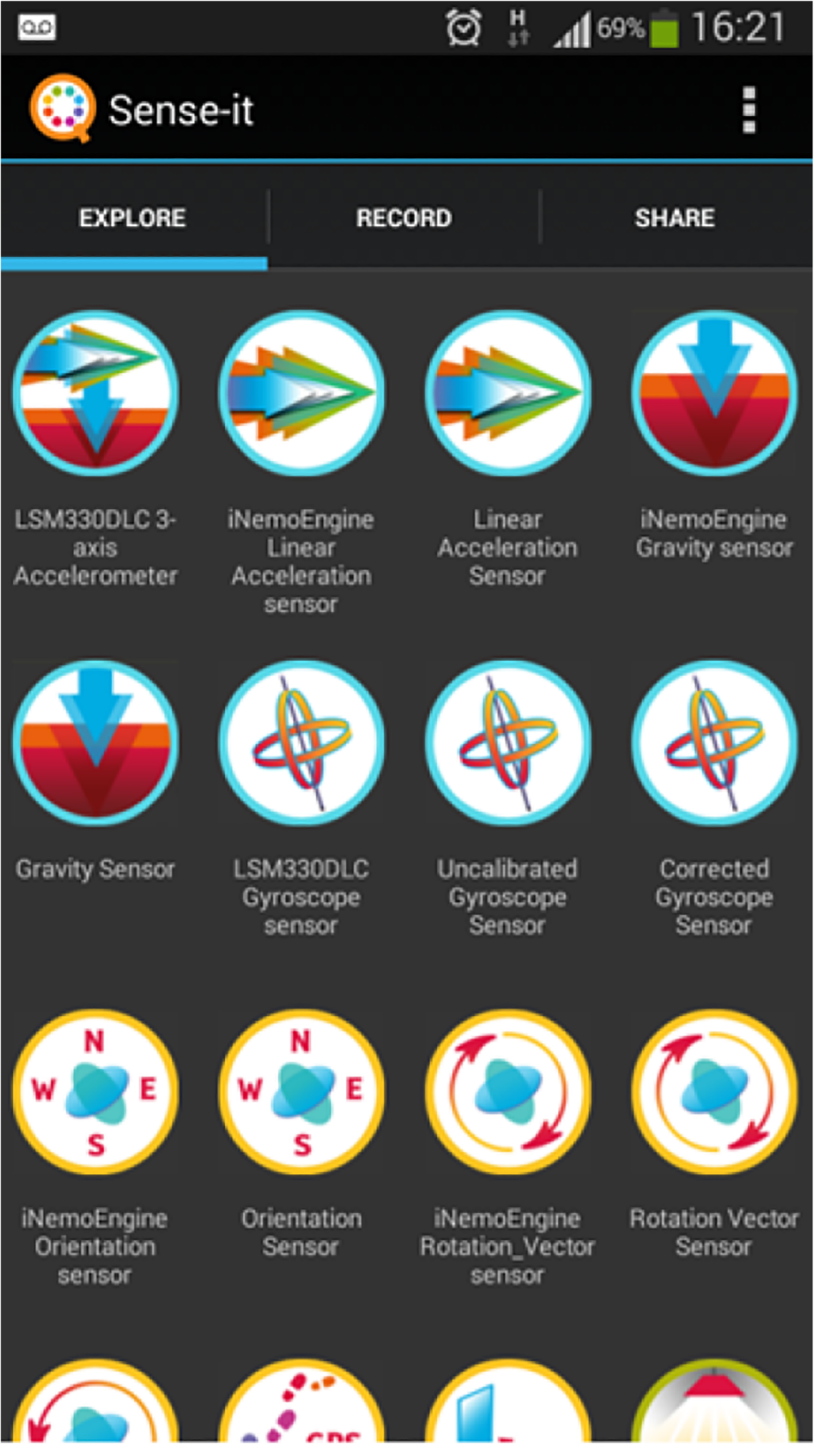
The Explore tab showing the number of sensors available on a given mobile Android device such as accelerometer, gravity sensor, and orientation sensor

**Fig. 2:**
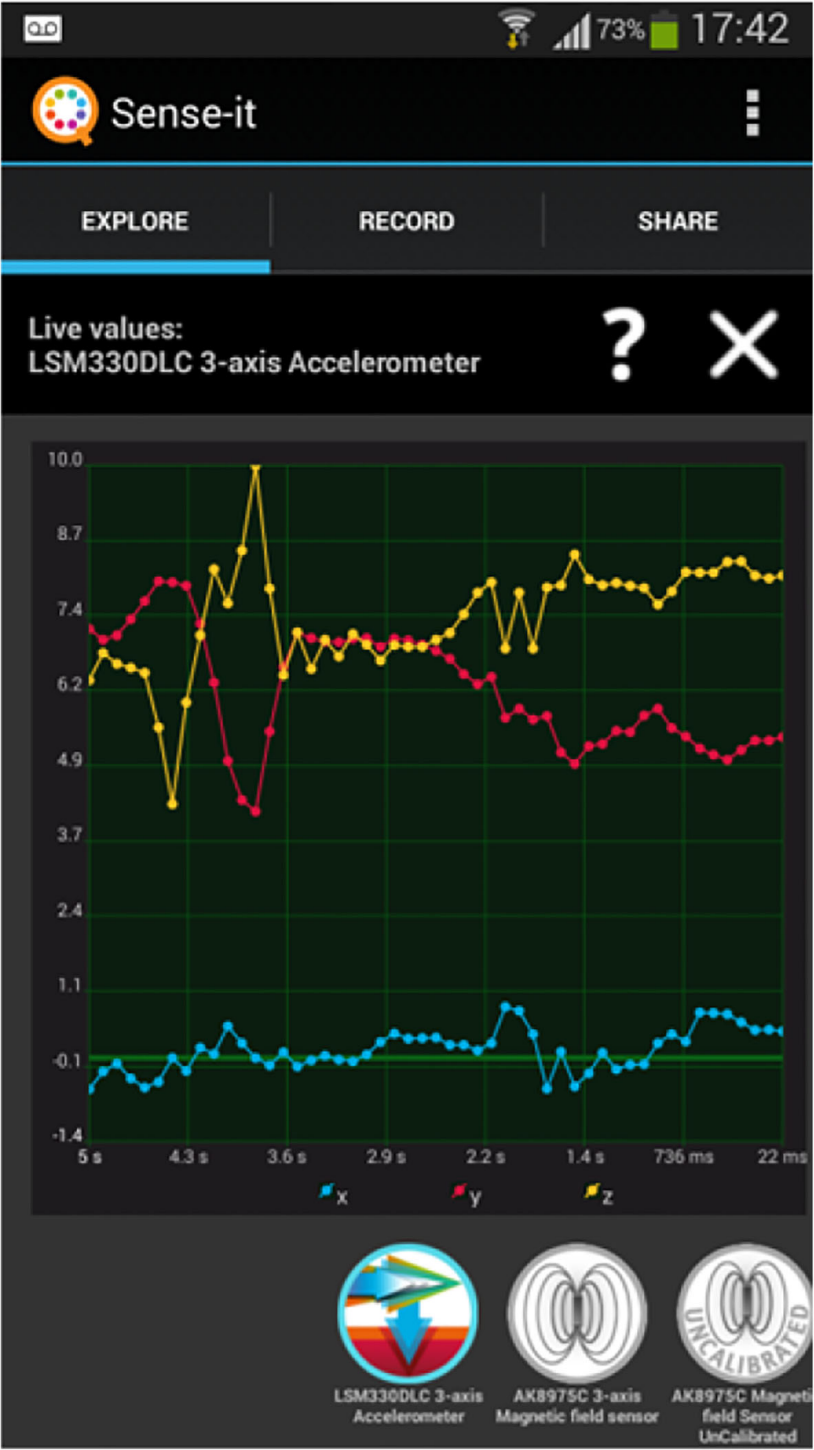
By selecting a sensor (e.g. accelerometer), a live recording of data is previewed

**Fig. 3:**
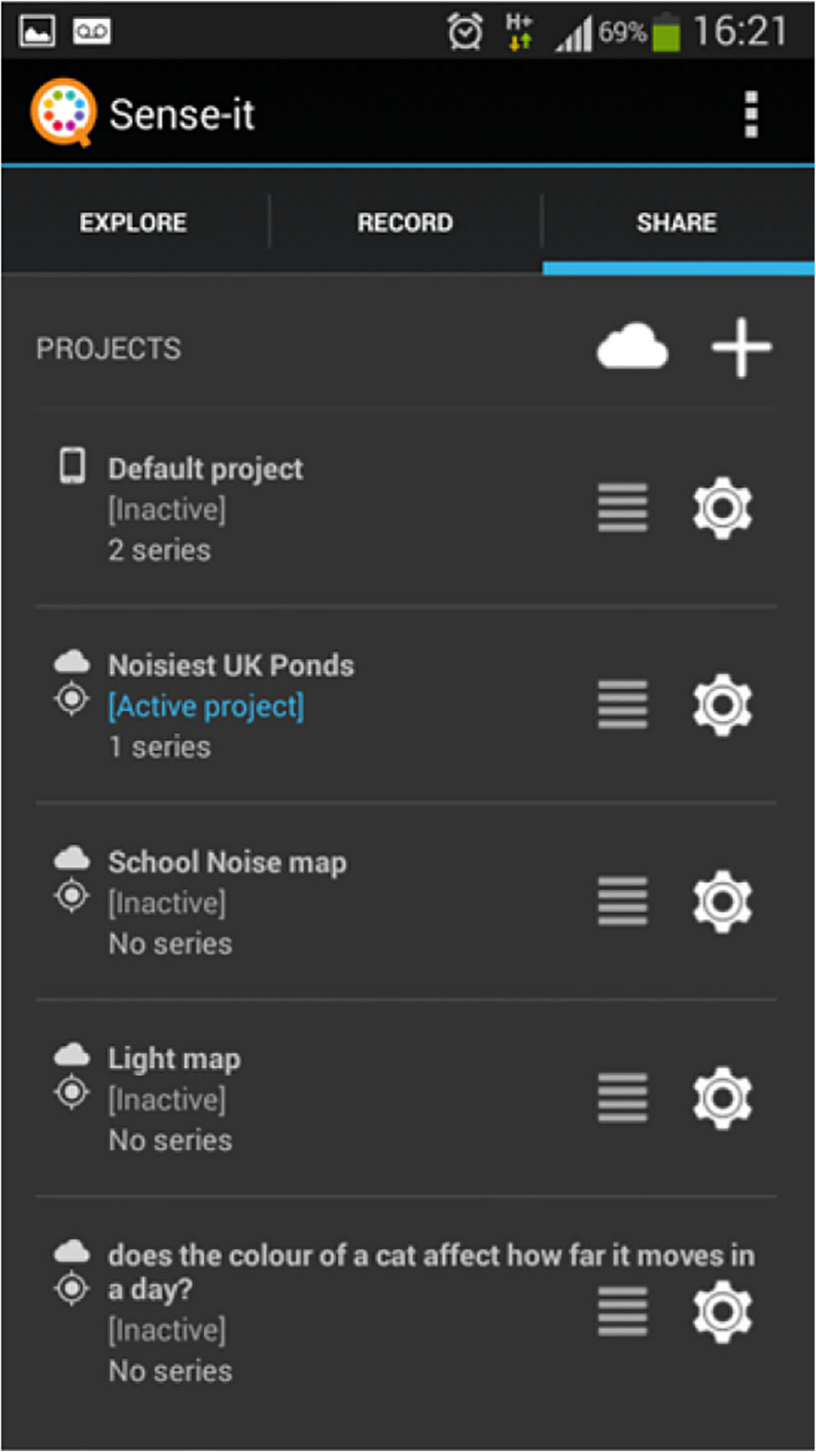
The Share tab showing the number of projects available to join. The selected/active project is the ‘Noisiest UK ponds’

Feedback from the three design experiments resulted in the design of the nQuire tools as presented below. Sense-it is a mobile application (see Figs. 1, 2, and 3) that gives access to sensors on phones and tablets—such as their accelerometer, light, sound, and humidity sensors—and allows users to capture, visualise, store, and download log files from these (Herodotou, Villasclaras Fernandez, Sharples, 2014a). Sensor-based applications designed for citizen science initiatives can be identified online, for instance, the iSPEX add-on to smartphone cameras delivering accurate data on dust particles in the atmosphere (Snik et al. 2014) and the NoiseTube to record environmental conditions (Maisonneuve et al. 2010). Their main functionality is restricted to capturing nature and wildlife and reporting on environmental conditions as a means to solve science-related problems. Also, they make use of a specific subset of sensors available on smartphones. In contrast, the Sense-it app allows users to make use of all the sensors available on a given mobile device, connects sensor recordings to a diverse set of citizen inquiry projects, provides instant visualisation of sensor recordings, and scaffolds users in proposing and designing their own citizen inquiry investigations. The Sense-it connects to the nQuire-it platform and enables users to download existing profiles and upload data to existing citizen science projects available on the platform.

**Fig. 4:**
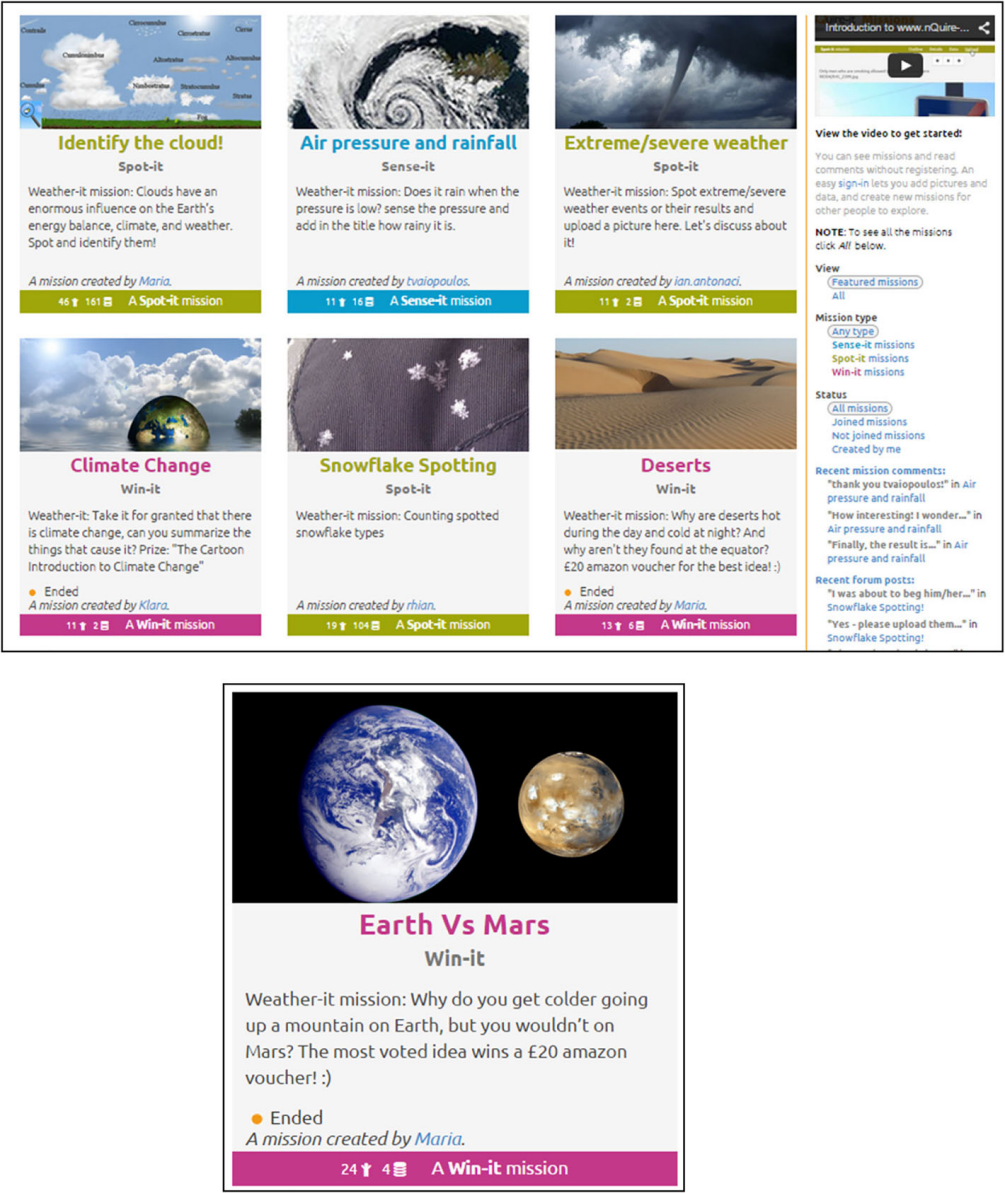
The three types of missions on the nQuire-it platform: Spot-it missions in green, Win-it missions in pink, and Sense-it missions in light blue

The nQuire-it Missions platform (www.nquire-it.org) enables non-professional members of the general public to engage in innovative inquiry-based learning through joining, proposing, conducting, and reporting inquiry-led scientific projects. The platform features three types of citizen inquiry projects, termed as ‘missions’ (see Fig. 4):

1. *Win-it missions* are challenge-based inquiries that offer rewards or prizes to the winners. Each challenge requires a creative response to an everyday problem or a scientific topic. For example, ‘propose an imaginative way to attract bumblebees to gardens’.
2. *Sense-it missions* make use of the Sense-it mobile app to collect and share data using mobile devices (see previous section). An example Sense-it mission is ‘where is the fastest lift located?’
3. *Spot-it missions* require users to capture and describe images using their phone camera, such as cloud formations or extreme weather.

The nQuire-it Missions platform supports three distinct types of interaction: (a) Users can view existing missions including mission details and data uploaded to missions without registration to the platform. They are expected to exhibit a minimum level of interest or curiosity to engage with the platform and explore its features and affordances. (b) Users register on the platform and contribute to one or more of the existing missions. Contributors are expected to comprehend the aims and methods of the mission they are contributing data to, make use of the right tools to collect data, upload their data to the mission, and interact with peers to conclude on the outcomes of the mission. Learning is self-directed and scaffolded by the nQuire-it toolkit and through interaction with peers. (c) Users create a new mission from scratch. Mission creators are required to engage with the platform over a longer period of time to understand the functionality of different types of missions, decide the type, topic and methods of their mission, describe adequately the mission for others to contribute data, and coordinate possible forum discussions and comments.

### Second design iteration with experts and end user interviews

The partnership with the BBC Tomorrow’s World initiative led to a second cycle of external critique of the nQuire tools, in particular the nQuire platform. This was a 4-week implementation initiated with a kick-off workshop with experts in technology-enhanced learning, online pedagogy, and user experience from the [name of university removed] (*N* = 8) and facilitated by a strategic design and innovation consultancy recruited to facilitate the process of redesign and development. The aim of the workshop was to define the goals of the nQuire platform, the intended audience, and the overall vision. The goals of the platform were defined as follows: the platform should facilitate structured curiosity, help people develop (through activity) the mindset and skills of a scientist, bring science into people’s lives, allow people to become part of a collective project (bigger than you could do by yourself), allow people to ask big questions, allow people to explore big ideas with others, collect large data sets to help answer big questions, foster belonging to a community through contributing, and create a portal for scientific inquiry.

Overall, these goals reflect the concept and definition of citizen inquiry, and in particular, the attempt to transform the relationships between environment and people as well as volunteers and scientists by challenging existing conceptions of knowledge production as emerging exclusively from scientists and the role of general public as being a consumer (rather than a producer) of this knowledge. Designing a platform that supports the aforementioned goals can or may influence as a result how people interact with technology and open up new expectations as to what the role of the ‘user’ should be in citizen science projects which could further shape the design of citizen science tools in the future. In accordance with these goals, intended audience was defined as leisure learners and hobbyists without necessarily having deep subject knowledge of a topic they are interested in exploring, amateur scientists, people with a curiosity about their world and with a social conscience, people with subject matter interest (over expertise), parents and children learning together, and topic experts wishing to use the data. The vision of the platform was to support members of the public to act as scientists and help them learn the ‘scientific method’ through participation and activity. This should be a human-centred participation platform in which people do and learn about science. Science is broadly defined to refer to both natural and social sciences and participation in citizen science projects across diverse domains and disciplines (Herodotou, Sharples, Scanlon, 2018).

In the follow-up weeks, the facilitator implemented activities to inform the design of the platform, including reviewing academic research relevant to the design of the platform (e.g. papers detailing the theoretical concepts behind the design of the platform, papers about digital design requirements in online platforms, and how they can support engagement, participation, and learning), reviewed the platform’s learning analytics (e.g. number of users, number of sessions) as well as similar science-related and community participation websites, and collected screenshots and ideas that could be discussed in the next workshop and potentially inform the redesign of the platform. These sources of information were used to develop a shared understanding of what it is known about designing citizen science platforms (theoretically and practically) and use this knowledge to inform the redesign of the platform. In addition, to get an insight into actual user motivations, needs, barriers, and areas of delight the facilitator carried out formative one-to-one interviews (*N* = 7). The structured conversations included both the existing nQuire-it platform and other websites with similar propositions. Four of the interviewees had used the current nQuire-it website in the past, and three of them were involved in other science participation projects. Interviewees shared their experiences of using citizen science platforms, including perceived benefits and challenges from participation.

### Findings from the second design iteration

**Fig. 5:**
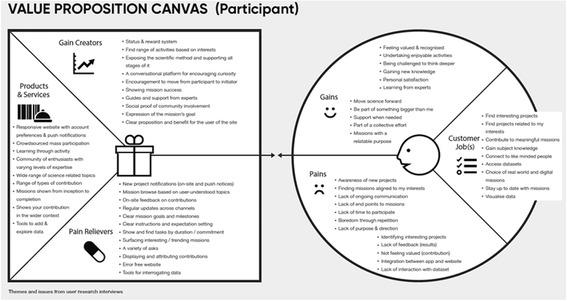
Interview themes from the perspective of a mission participant

**Fig. 6:**
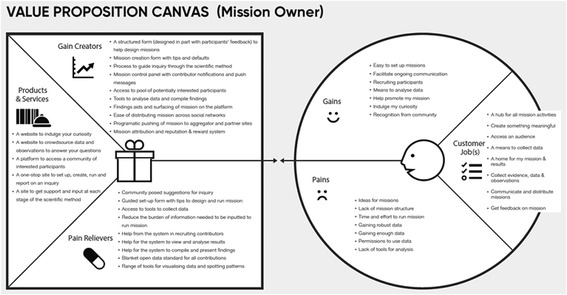
Interview themes from the perspective of a mission owner

Interview data were clustered around main themes as detailed on a value proposition canvas (Jobs, Gains, Pains, see below). A value proposition canvas is a diagram that captures and shares the research outputs and provides suggestions as to how the insights can be taken forward into the redeveloped website. The canvas was used to organise and plot interview data. Figure 5 shows the outcomes from the perspective of a mission participant, and Fig. 6 shows the outcomes from a mission owner perspective. The right side of the canvas is the circular user or customer profile, and it is divided into three sections: ‘Customer Jobs’ which describe what the user is trying to get done, ‘Gains’ listing the outcomes and goals of the audience, and ‘Pains’ stating barriers and obstacles faced. The left side is the square value map. It highlights ways of addressing the themes raised in the customer profile.

Summarising the design requirements from the perspective of a participant in a citizen inquiry project, interviewees expressed the need for projects relevant to their interests that could connect them to like-minded individuals and that would help them learn (see Customer Jobs). As an interviewee explained: ‘I found a passion in citizen science it gave me something meaningful to contribute to’. In terms of gains, participants’ expectations were either personal or catholic; they seek to enjoy participation in citizen science projects and gain certain cognitive and affective benefits such as recognition, satisfaction, deep thinking, and knowledge acquisition. As stated: ‘a good website challenges me to think a little deeper’. In addition, more universal expectations were expressed such as moving science forward and be part of a collective effort. As explained: ‘You need to convince me it will be useful to science or I’ll keep uploading my images to Instagram instead’. In terms of barriers or obstacles faced when participating in existing citizen science platforms, they raised issues related to the design of the mission such as length and topic of tasks and lack of feedback and support to guide interactions. As explained: ‘Time is important both how long will it take to do the tasks and when will I get to see some results?’ and ‘You should leave the community with some analysis to make people feel their contribution is worthwhile’.

These insights were translated into design recommendations (see Fig. 5) that could be taken forward to inform the redesign of the new platform and mapped around: (a) products and services including mechanisms to indulge curiosity, crowdsourcing of data to answer questions, access to a community of interested participants, creation and reporting on a mission, and ongoing support at each stage of the scientific method; (b) gain creators including forms to easily structure the design of new missions, procedures to guide inquiry through the scientific method, notifications and push messages, tool to analyse and merge data, distribution of missions across platforms, and reputation and reward system; and (c) pain relievers including mission ideas suggested by the community, access to tools to collect and visualise data, system support to recruit contributors to a mission, system support to view and analyse data, and open data standard for contributions.

*Design principle 5: Communicate key message of learning by doing.*

*Design principle 6: Communicate key messages of ‘doing and being part of a community’*

*Design principle 7: Reward every visit to the citizen inquiry platform*.

Summarising the design requirements from the perspective of a mission owner (see Fig. 6), interviewees expressed the need for a hub hosting all mission-related activities, tools to collect data and host results, communication and mission feedback from others, and ways to distribute missions. As stated: ‘Reaching participants is a headache for researchers that the platform could solve’. In terms of gains, they expressed the need for an easy and straightforward way to set up missions, ways to recruit participants, and promote missions as well as mechanisms to get recognition from the community. In terms of obstacles or challenges, they raised issued related to a lack of ideas as to what mission to set up, lack of mission structure and time to create and manage a mission, gaining enough and valid data, and the need for tools to support data analysis. These data were translated into design principles related to (a) products and services such as a one-step site to set up, run, and manage missions and a portal to access a community of interested participants; (b) gain creators such as structured forms to help mission set up including defaults and scaffolds to guide the processes of scientific inquiry; and (c) pain relievers such as community suggestions for missions, tools to recruit contributors, collect, analyse, and visualise data.

*Design principle 8*: *Value users and their time.*

## Discussion

The design-based research (DBR) methodology adopted in this 4-year study led to the production of a set of requirements for designing citizen inquiry projects which are applicable to interaction design work and of relevance to researchers aiming to explicitly scaffold inquiry learning in citizen science projects. These requirements resulted from the iterative evaluation of citizen science tools with end users through three design experiments, seven structured interviews with users of citizen science and community platforms, and ongoing consultancy by a group of experts in technology-enhanced learning, online pedagogy, and user experience.

Design principles emerging from the first external design critique:

- *Design principle 1 (theory): Engage volunteers as initiators of citizen science projects, to create personally relevant investigations*. Aligning with the concept of citizen inquiry, nQuire-it allows users to set their own research objectives and create different types of missions. The ‘Create’ tool allows users to set a title for their mission, provide instructions on how to join the mission and add data, and customise it by adding any other additional information. In the case of Sense-it missions, they define the sensor profile of a mission by deciding the set of sensors needed for data collection, the sampling rate as well as how data will be processed when uploaded to the platform.
- *Design principle 2 (theory): Vary the investigations (types and topics) to accommodate different interests and motivations of volunteers who participate in citizen science projects*. By acknowledging that people have very different interests and motivations for engaging in citizen science, projects with multiple ways to participate are more likely to reach larger and broader audiences (Bonney et al. 2016). The nQuire-it platform offers a variety of missions (investigations) that vary thematically. For instance, the missions created within the Weather-it project attempt to provide answers to weather-related questions emerging from participants’ everyday experiences such as ‘How does the colour of the sky change at sunset?’, shared concerns such as climate change, or phenomena participants are curious about such as extreme weather conditions. Other nQuire-it missions created by participants relate to energy use (e.g. ‘green’ cars), ecology and environment (e.g. bee extinction, how city noise might affect birds), and astronomy (e.g. proposing astronomy investigations using a real telescope).
- *Design principle 2 (revised): Topics before tools*. This principle was revised after consultation by experts in the second cycle of design critique and user experience feedback from design experiments showing that it was difficult for users to set up tool-based investigations as they could not grasp how tools should be used to set up specific investigations. By setting topics over tools, the platform aims to give users routes to find content of interest to them. This will allow discovery based on terms known to the users. Surfacing topics in the interface will also let express the scope and offer of the website.
- *Design principle 3 (user experience): Develop mobile applications to scaffold data collection citizen inquiry projects*. Despite their worldwide diffusion, portability, and ease of use, mobile devices are not yet used to their full potential in learning applications to measure and investigate real-world phenomena (Herodotou, Villasclaras Fernandez, Sharples, 2014a). Our design experiments with young people revealed users’ satisfaction with using personal mobile devices to run scientific investigations as well as using mobile applications to collect data in particular from mobile sensors (Herodotou, Villasclaras Fernandez, Sharples, 2014a, 2014b).
- *Design principle 4a (user experience): Scaffold the process of scientific inquiry from setting up a new investigation, to choosing tools for data collection or contributing to investigations set by others.*
- *Design principle 4b (user experience): Moderate interactions and facilitate participation through a set of mechanisms such as weekly email notifications*. As illustrated in the functionality of nQuire-it and theoretically in the conception of citizen inquiry, learners define and direct their own learning through a range of tools. To support learning, scaffolding mechanisms were integrated into the design of nQuire-it, in particular to assist the process of setting up a citizen inquiry and data collection practices (Herodotou, Villasclaras-Fernandez, Sharples, 2014b). Visual conceptual organisers represent the basic operations of science inquiry, including naming and describing a mission, setting objectives, providing guidelines on how to take part in missions, and selecting the methods of data collection using the Sense-it app, a phone camera, or text input. Future development plans include moderators or facilitators who will be in charge of engaging with participants by, for example, emailing them with platform notifications or resolving forum inquiries. Their role will be to keep participants engaged with the platform activities.

Design principles emerging from the second external design critique:

- *Design principle 5: Communicate key message of learning by doing*. Elements in the interface of the website will communicate the key messages of ‘doing activity’ (and learning through it) and ‘being part of a community’ (see Design principle 6). The platform will support learning by doing by supporting users through the stages of the scientific method and helping them become more structured in their thinking. The primary means to achieve this will be through encouraging activity. The core invitation to users is to participate, create and facilitate inquiry, and not just contribute with their data.
- *Design principle 6: Communicate key messages of ‘doing and being part of a community’*. The platform will allow a wide community to indulge their curiosity and to develop their skills, knowledge, and confidence. As interview data suggested, users are more likely to use and keep using the website if they see and feel behind it is a vibrant community achieving a purpose. To highlight the active community, the platform will promote conversation and action on all items, dynamically surface popular and changing content, show the people behind the contributions, and use the audience to build the future audience.
- *Design principle 7: Reward every visit to the citizen inquiry platform*. However, short or long a user is on the platform, whether it is a first or a repeat visit, the website will provide a rewarding experience. Ideally, this reward will be one or a mixture of learning something, engagement with the community, making a new discovery, or encouragement to participate.
- *Design principle 8*: *Value users and their time*. Success of any community platform comes from ongoing engagement and activity of users. To respect and reward users’ time, the platform will ensure to maximise the flow around the site and in undertaking tasks, make it easy to understand and to use, and provide feedback for participation.

## Issues and limitations

The creation of a sustainable community of inquiry is one of the major challenges of citizen inquiry. An increase in the number of nQuire-it sessions per week was observed in year 2, more likely due to the moderation and mentoring of the activity by a Ph.D. student that encouraged participation. This activity declined rapidly by the end of the project suggesting that engaging facilitators to monitor the online activity and scaffold different missions can support participation and sustainability. Participation and community creation could be further enhanced by mechanisms including a reputation system. Reputation systems provide external feedback and recognise and reward specific activities the system asks people to engage with. The iSpot system is a successful example of such an implementation. Offering online badges to experts and social and scientific scores (i.e. scores are obtained when uploading and identifying an observation or when a member agrees with a given identification) facilitated the evolution and maintenance of a large community (Clow and Makriyannis 2011). Another web-based application that has successfully adopted such mechanisms is Stack Overflow (http://stackoverflow.com/), a question and answer website for professional programmers. In social games such as Farmville, a planting and harvesting crops game, certain accomplishments are rewarded with badges which in turn unlock new game challenges. To promote social recognition and blur the boundaries between gaming and social network sites, special rewards are received when badges are shared socially in networks such as Facebook (Landers and Callan 2011). These examples demonstrate the power of reputation systems to engage users with specific activities and reinforce ongoing participation which is an essential requirement for forming an online learning community. Further to that, it is our intention to engage more users with the platform and initiate the development of a sustainable online community through mechanisms such as (a) the recruitment of ‘young ambassadors’ who would systematically engage with activities on the platform and share news and missions with their social networks, (b) communication with schools to identify ways of using the tools in science teaching and learning, (c) blogging in specialist education press, (d) publication of ‘success stories’ in the form of case studies and analytics indicating depth of engagement, and (e) participation in events to showcase the tools to the wider public.

In addition, citizen inquiry faces the challenge of opening up investigatory science to people without prior training in scientific methods. To address this challenge, we intend to recruit expert scientists from academia, science organisations, and schools as regular participants on the platform to guide newcomers through the inquiry process by proposing sample missions, reviewing the quality of new missions and collected data, and by scaffolding discussions and argumentation. This approach was successfully adopted in the case of Weather-it missions; weather experts were recruited from weather and science engagement organisations, joined the nQuire-it platform, and evaluated the content, methods of data collection, and contributions provided by less expert members. Legitimate peripheral participation comprises a central process in community maturity (see Lave and Wenger 1991). New members of the community will initially join existing missions or create simple and not well-elaborated ones, in terms of content and quality. Through communication with other users and feedback received by experts, they will familiarise themselves further with the inquiry process, robust methodologies for data collection, and analysis and assessment of the quality of contributions. As their participation increases and their understanding of the processes and principles of the community enhances, it is anticipated that they will become more central members of the community and support novices to move from periphery to the centre.

Another challenge relates to the use of the Sense-it app and sensor calibration. Trials to test the calibration of light sensors on mobile phones showed a wide divergence of measurements that urged to further investigation. To this end, a variety of factors that may affect the sensor measurements were revealed, such as the brand/model of the device, the type of sensor (e.g. linear), the existence of maximum values, the percentage tolerance, and hardware damages (Aristeidou, M., Scanlon, E., Sharples, M., 2015a). The risk of unreliable measurements could be turned into an opportunity for learning how to calibrate the sensors in mobile devices used for scientific inquiries, by using, for instance, professional sensors located in the recorders’ area (e.g. from local weather stations for air pressure) to retrieve more valid values.

## Conclusions

In this paper, we detailed the design of a set of citizen science tools for supporting inquiry learning, and in particular, how pedagogy-led design and user experience studies can be used to inform the development and ongoing iteration of citizen science tools. Our user studies revealed what end users expect from interacting with tools and what their requirements are for engaging and participating in citizen inquiry projects. We used a design-based research methodology to guide the design of web-based and mobile citizen science tools which are theoretically grounded on the innovative concept of citizen inquiry and the engagement of the general public in initiating personally meaningful scientific investigations. To achieve this, we designed a management system for the creation of citizen inquiry projects and mobile tools to assist the process of data collection, visualisation, and analysis. While other citizen science projects are found to entail elements of educating the public, none of these approaches is found to do this explicitly and systematically. The nQuire-it toolkit is a step in this direction which at the moment is undergoing developments to effectively scaffold members of the public in understanding and engaging with all the stages of the scientific method.

The long-term and iterative process of designing digital artefacts around citizen inquiry led to the production of a set of design constructs, specifically the identification of design requirements to guide the design of online citizen inquiry projects. These are summarised in eight design guidelines: users as producers of knowledge, topics before tools, mobile affordances, scaffolds to the process of scientific inquiry, learning by doing as key message, being part of a community as key message, every visit will bring a reward, and value users and their time. These requirements are a major starting point for guiding the interaction design of citizen inquiry projects. Further studies aim to evaluate the redesign of the tools and provide evidence as to whether and how users learn from interacting with the tools and the community while further user experience evaluation studies will refine how these principles manifest in tools’ design.

Our initial studies of the nQuire-it platform have raised issues of how to enable people to frame valid inquiries, how to engage scientists in these endeavours, and how to sustain an active community of citizen inquiry. In terms of the latter, a sustainable community is essential in order to scaffold the learning journey of novice members who are not familiar with the platform and its affordances and maintain the interest and participation of more expert members in the online activities of the community. Our partnership with the BBC Tomorrow’s World initiative and relevant platform publicity are expected to spread the word across diverse audiences and contribute to developing an active community of individuals around the platform.

Considering these challenges, we have plans to develop further the nQuire-it toolkit. This will be done by adding a reputation system to reinforce ongoing participation and engagement with missions and designing other mobile applications such as a Spot-it app to facilitate data collection and upload to Spot-it missions using mobile devices. It will be important to recruit scientists and young ambassadors to provide expert advice to novice users and spread the word around missions in their social network sites accordingly and explore venues for collaboration with existing online citizen science projects to enable the initiation of inquiry projects using existing citizen science databases. In addition, the intention is to introduce the nQuire-it toolkit to the school community as a teaching and learning tool that can bridge the gap between formal and informal learning practices when used, for instance, in a flipped classroom learning context (e.g. Herreid and Schiller 2013) or more traditional classroom conditions with students defining and implementing their own missions supported by more expert individuals, their teachers. The premise behind the design of the nQuire-it toolkit and the conceptualization of citizen inquiry is to equip the general public with the required tools for self-directed lifelong learning experiences.
